# Supplementary material for: Early alveolar epithelial cell necrosis is a potential driver of COVID-19-induced acute respiratory distress syndrome
Source: iScience. 2022 Dec 6;26(1):105748. doi: 10.1016/j.isci.2022.105748 (PMC9722615; doi:10.1016/j.isci.2022.105748)
Supplement: Document S1. Figures S1-S9 and Tables S1-S3 [file mmc1.pdf]

iScience, Volume *26*

Supplemental information

Early alveolar epithelial cell necrosis

is a potential driver of COVID-19-induced

acute respiratory distress syndrome

Kentaro Tojo, Natsuhiro Yamamoto, Nao Tamada, Takahiro Mihara, Miyo Abe, Mototsugu Nishii, Ichiro Takeuchi, and Takahisa Goto

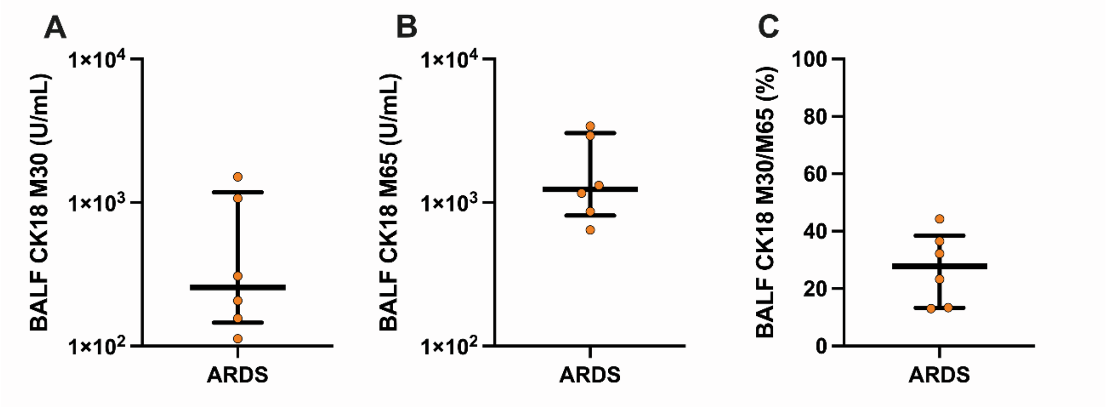

Figure S1. Levels of epithelial cell death markers in BALF samples, Related to Figure 2.

Levels of (A) CK18-M30, an epithelial apoptosis marker; (B) CK18-M65, an epithelial total cell death marker; (C) CK18-M30/M65 ratio, an indicator of the fraction of epithelial cells undergoing apoptosis versus all types of cell death in BALF samples of COVID-19 patients with ARDS

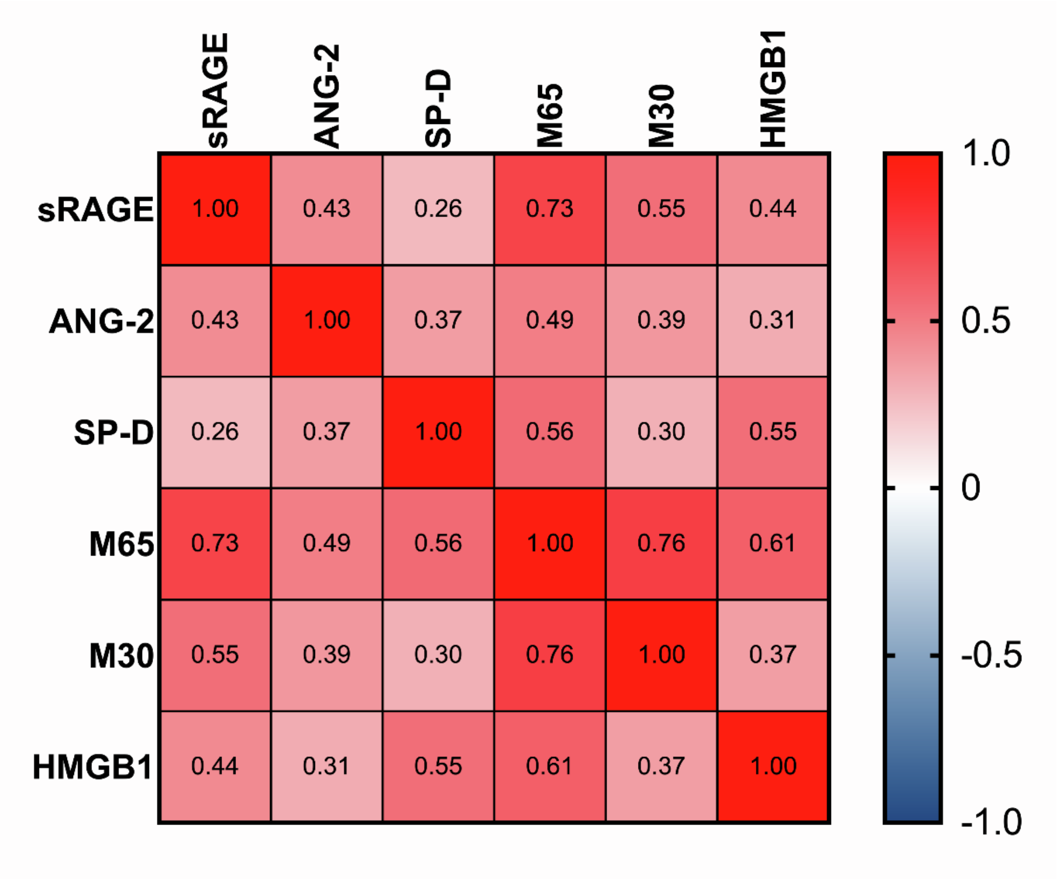

Figure S2. Pearson correlation coefficients among circulating markers, Related to Figure 1 and 2. The serum levels of each marker were log-transformed and then Pearson's correlation analyses were performed.

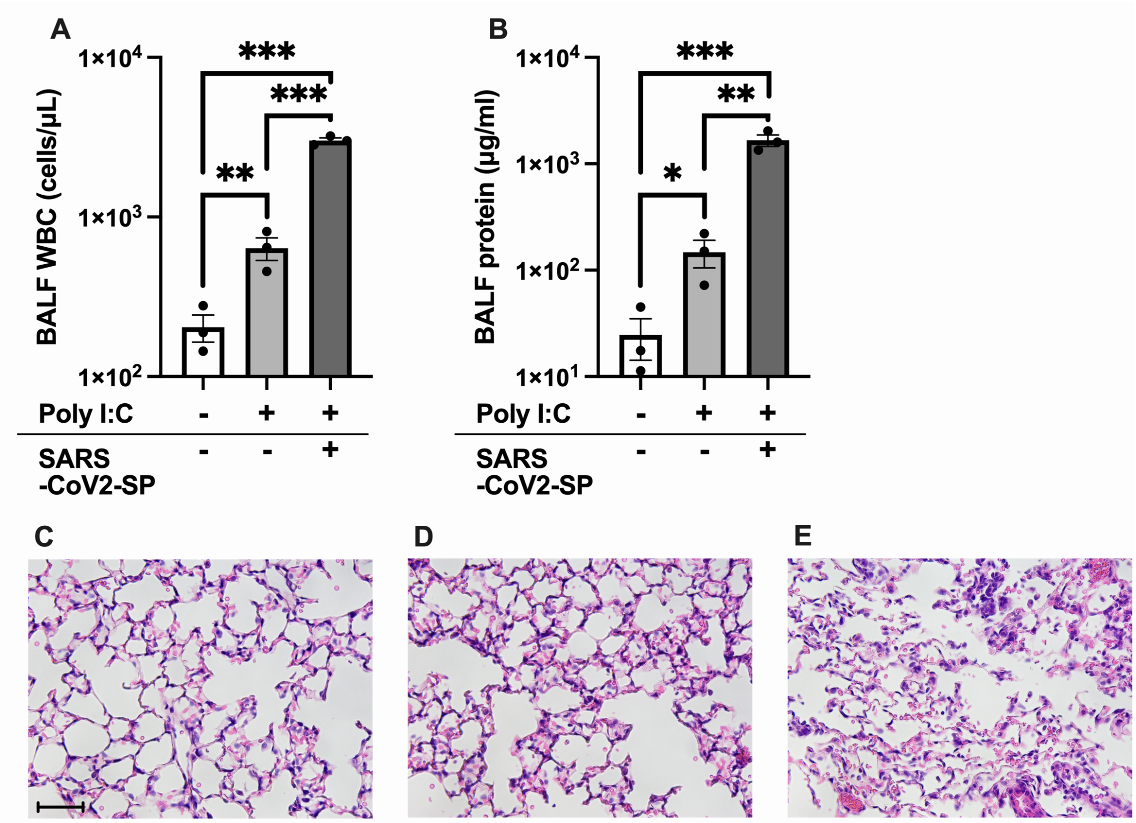

Figure S3. Effects of intratracheal administration of the severe acute respiratory syndrome coronavirus 2 (SARS-CoV-2) spike protein combined with polyinosinic:polycytidylic acid (poly (I:C)), Related to Figure 3. Levels of (A) white blood counts and (B) total proteins in the bronchoalveolar lavage fluid of mice intratracheally injected with phosphate buffered saline, poly (I:C) or SARS-CoV-2 spike proteins combined with poly (I:C) are shown. (C) Representative images of lung tissue sections stained with hematoxylin and eosin. Scale Bar = 50μm. The values are presented as means ± standard error. *$p<0.05$, **$p<0.01$, ***$p<0.0001$

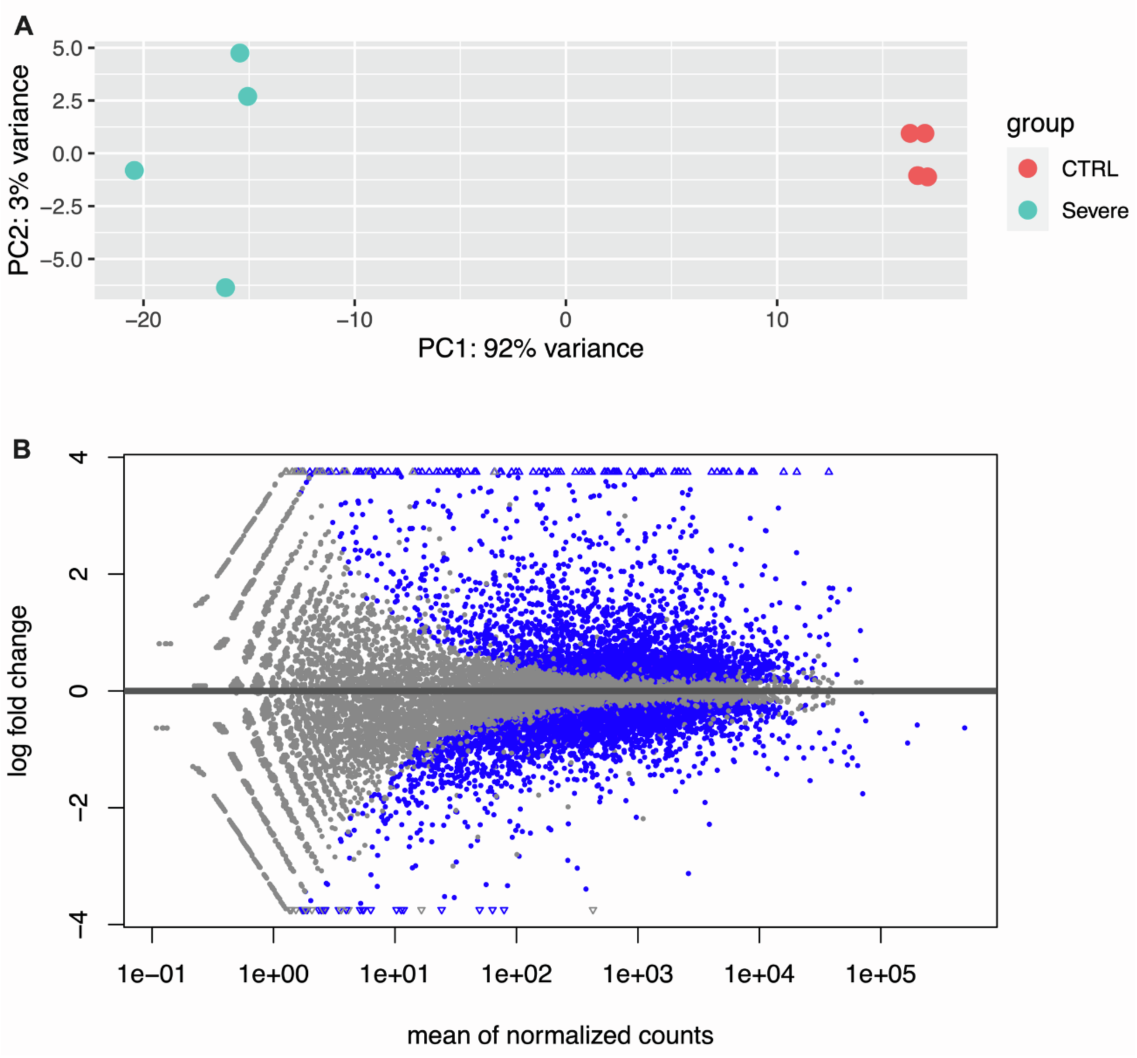

Figure S4. Analysis of RNA-seq data of the COVID-19 model using SARS-CoV-2 spike protein and poly (I:C) in the present study, Related to Figure 4. (A) Principal component analysis and (B) MA-plot comparing the severe COVID-19 model and the control

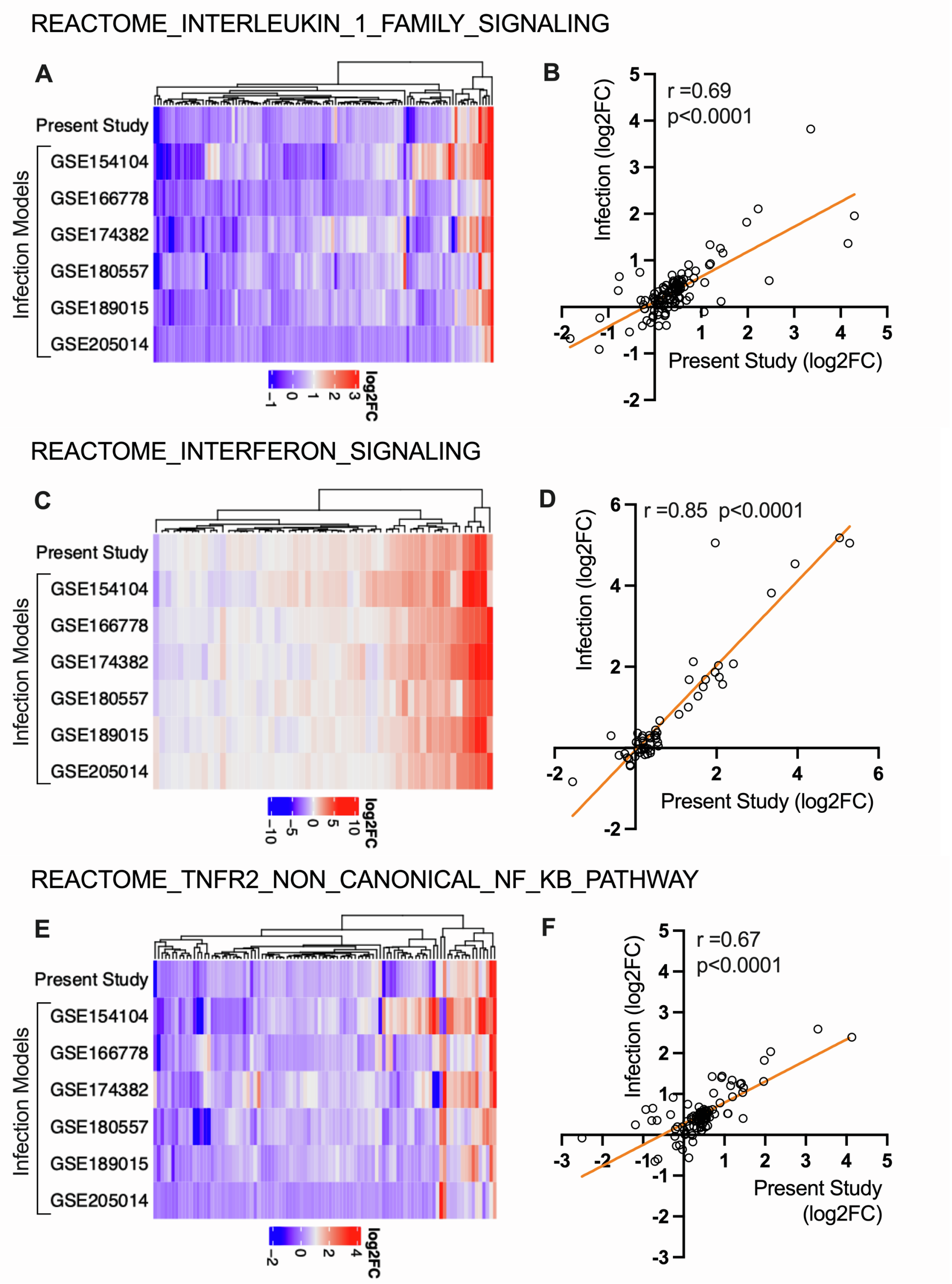

Figure S5. Analysis of gene expression patterns in key inflammatory pathways, Related to Figure 4. (A, B) Interleukin-1 family signaling, (C, D) interferon signaling, and (E. F) TNFR2 non-canonical NFκB pathways in the REACTOME database. Heatmaps of gene expression patterns (A, C, and E) and correlation analysis between the log_2 fold-changes in the present study model and mean log_2 fold-changes in the infection models (B, D, and F) are shown

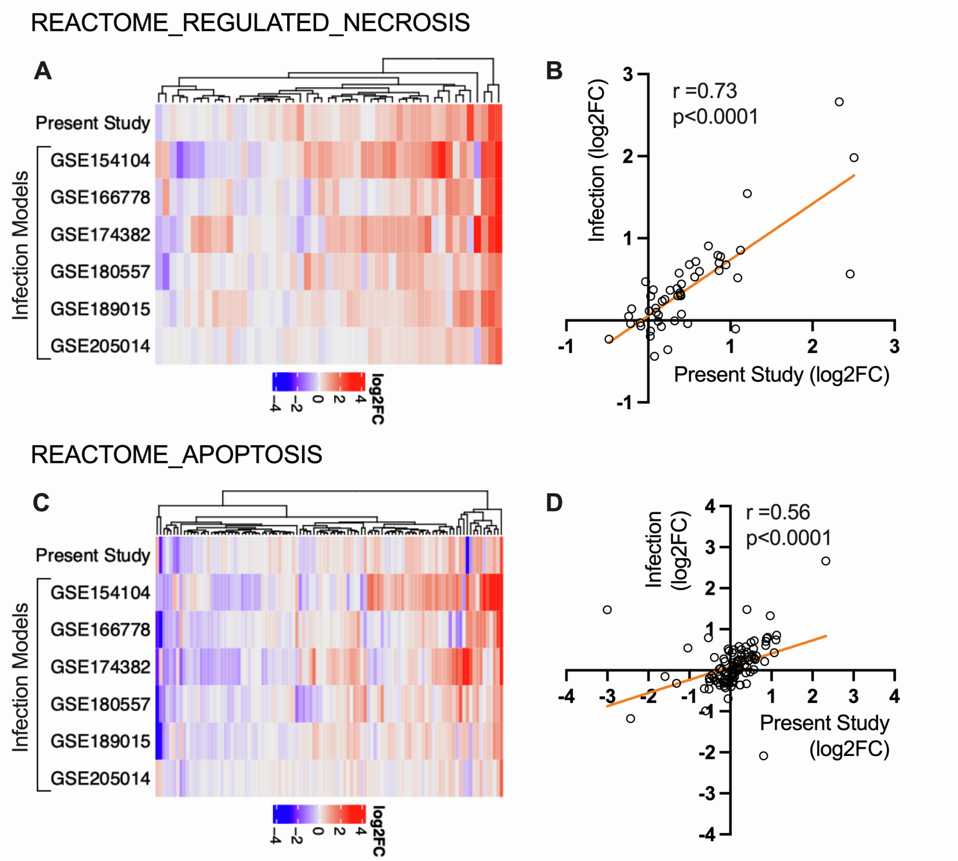

Figure S6. Analysis of gene expression patterns in programmed cell death pathways, Related to Figure 4. (A, B) Regulated necrosis, and (C, D) apoptosis pathways in the REACTOME database. Heatmaps of gene expression patterns (A and C) and correlation analysis between the $\log_2$ fold-changes in the present study model and mean $\log_2$ fold-changes in the infection models (B and D) are shown

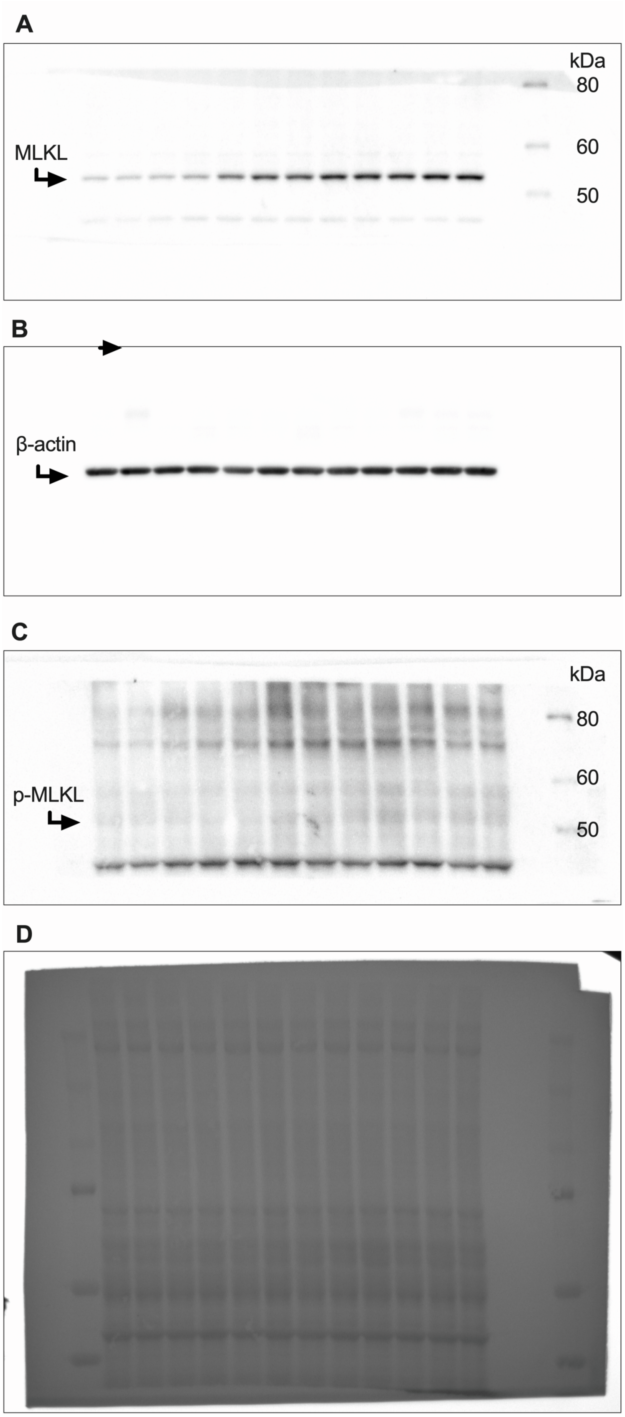

Figure S7. Uncropped images of immunoblots of (A, B) mixed lineage kinase domain-like (MLKL) and (C, D) phosphorylated MLKL, Related to Figure 5. Equality of protein loading was confirmed by (B) beta-actin staining (MLKL) after quenching horseradish peroxidase using hydrogen peroxide or (D) total protein staining (p-MLKL).

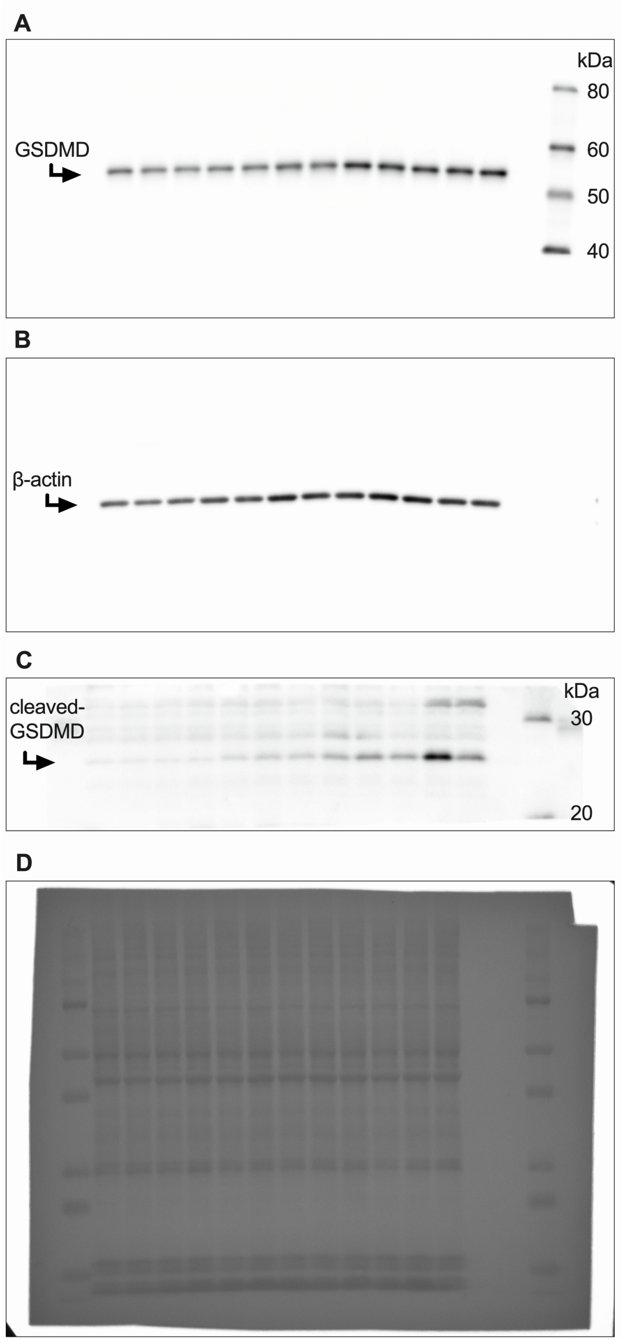

Figure S8. Uncropped images of immunoblots of (A, B) gesdermin D (GSDMD) and (C, D) cleaved GSDMD, Related to Figure 5. Equality of protein loading was confirmed by (B) beta-actin staining (GSDMD) after quenching horseradish peroxidase using hydrogen peroxide or (D) total protein staining (cleaved GSDMD).

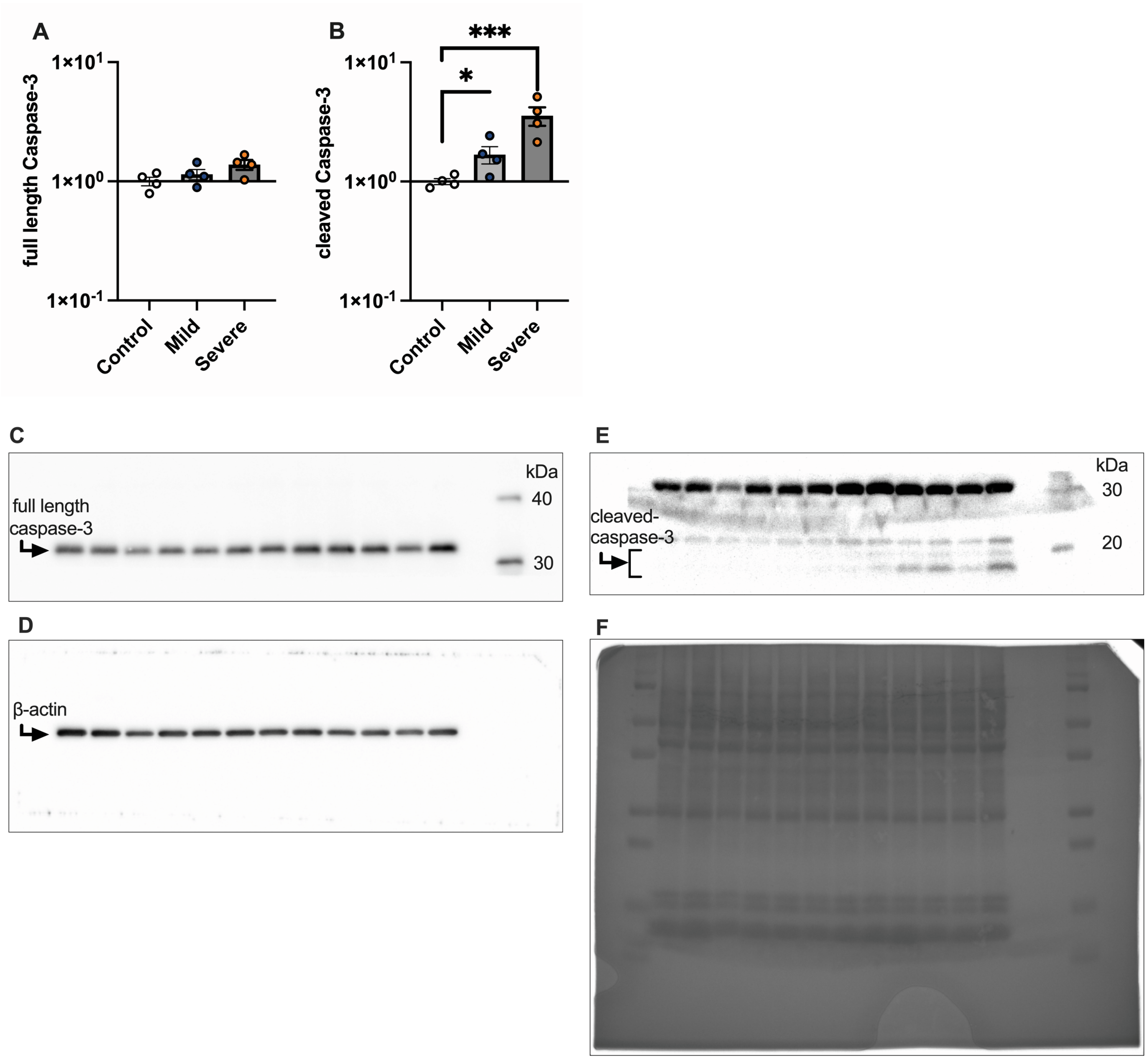

Figure S9. Immunoblot analysis of full length and cleaved caspase-3, Related to Figure 5. (A, B) Densitometry and (C-F) uncropped images of full length and cleaved caspase-3 immunoblots of the protein extracted from the lung tissue of a COVID-19 mouse model. Equality of protein loading was confirmed by (D) beta-actin staining (full length caspase-3) after quenching horseradish peroxidase using hydrogen peroxide or (F) total protein staining (cleaved caspase-3).

**Age (years)**	58 (55-71)
**Males/Females**	5/1
**APACHE2 score**	14.0(10.0-15.5)
**P/F ratio at admission**	154.5 (132.0-215.2)
**Mechanical ventilation use**	6 (100.0%)
**In hospital mortality**	1 (16.7%)
**Laboratory data on admission**	
**WBC count (/µL)**	8950 (3300-13700)
**Lymphocyte count (/µL)**	563 (435-916)
**Platelet count (×10^3/ µL)**	165.5 (110.3-195.0)
**D-dimer (µg/mL)**	1.30(1.14-3.32)
**CRP (mg/dL)**	4.69 (3.73-17.12)
**Creatinine (mg/dL)**	0.81 (0.57-4.23)
**Total bilirubin (mg/dL)**	0.40 (0.28-0.45)

Table S1. Clinical characteristics in ARDS patients (n=6) with COVID-19 whose BALF samples were collected, Related to Figure 2 and Figure S1. Data are presented as count (%) or median (IQR).

Accession Number	Strain	Age	Sex	Disease Tissue	Control Tissue
GSE216644 (The Present Study)	C57BL/6J	8 weeks	Male	Instillation of SARS-CoV-2 spike protein and poly(I:C)	Instillation of Phosphate-buffered saline
GSE154104	K18-hACE2	8 weeks	Male/ Female	SARS-CoV-2-infected lung	Non-infected lung
GSE166778	BALB/c	6 weeks	Male	Mouse-adapted SARS-CoV-2-infected lung	Non-infected lung
GSE174382	K18-hACE2	8-10 weeks	Male	SARS-CoV-2-infected lung	Non-infected Lung
GSE180557	K18-hACE2	8-16 weeks	Male	SARS-CoV-2-infected lung	Non-infected Lung
GSE189015	CD-1	8-10 weeks	Female	SARS-CoV-2-infected lung	Non-infected Lung
GSE205014	K18-hACE2	9 weeks	Female	SARS-CoV-2-infected lung	Non-infected Lung

Table S2. Details of the mouse models of COVID-19 datasets, Related to Figure 4.

	Present Data	GSE 154104	GSE 166778	GSE 174382	GSE 180557	GSE 189015	GSE 205014
**Up DEGs**	3487	674	3145	4825	3219	1712	520
**Dn DEGs**	3174	305	2976	4204	2910	1318	174
**Overlapped DEGs**							
**Present Data**		510 (14.0%)	1203 (22.2%)	1793 (27.5%)	1690 (33.7%)	837 (19.2%)	423 (11.8%)
**GSE 154104**	154 (4.6%)		388 (11.3%)	475 (9.5%)	445 (12.9%)	347 (17.0%)	259 (27.7%)
**GSE 166778**	817 (15.3%)	136 (4.3%)		1457 (22.4%)	929 (17.1%)	936 (23.9%)	316 (9.4%)
**GSE 174382**	1361 (22.6%)	146 (3.3%)	1374 (23.7%)		1433 (21.7%)	1240 (23.4%)	343 (6.9%)
**GSE 180557**	1471 (31.9%)	156 (5.1%)	746 (14.5%)	1370 (23.9%)		785 (18.9%)	365 (10.8%)
**GSE 189015**	372 (9.0%)	46 (2.9%)	635 (17.4%)	782 (16.5%)	458 (12.1%)		290 (14.9%)
**GSE 205014**	96 (3.0%)	13 (2.8%)	40 (1.3%)	55 (1.3%)	55 (1.8%)	31 (2.1%)	

Table S3. Overlap of differentially expressed genes (DEGs) among mouse COVID-19 models, Related to Figure 4.
